# Component-based CT radiomics for preoperative differentiation of well-differentiated and dedifferentiated retroperitoneal liposarcoma

**DOI:** 10.3389/fonc.2026.1924254

**Published:** 2026-09-09

**Authors:** Ke Liu, Junxiang Zhang, Yaru Ren, Haochen Wang, Shuting Yang, Ziyang Xue, Yanjun Sun, Ting Liang, Xuqi Li, Shufeng Wang

**Affiliations:** 1 Department of General Surgery, The First Affiliated Hospital of Xi’an Jiaotong University, Xi’an, Shaanxi, China; 2 Department of Radiology, The First Affiliated Hospital of Xi’an Jiaotong University, Xi’an, Shaanxi, China

**Keywords:** computed tomography, machine learning, preoperative differentiation, radiomics, regional radiomics, retroperitoneal liposarcoma

## Abstract

**Introduction:**

Preoperative distinction between well-differentiated liposarcoma (WDLPS) and dedifferentiated liposarcoma (DDLPS) remains difficult on conventional imaging. We evaluated whether CT attenuation-defined regional radiomics provides additional discriminatory information beyond whole-tumor radiomics and simple quantitative CT measurements.

**Methods:**

This retrospective study included 97 patients with surgically confirmed retroperitoneal liposarcoma (57 WDLPS and 40 DDLPS) who underwent preoperative contrast-enhanced CT. Radiomics features were extracted from the whole tumor (W), CT-defined fat-attenuation (F) and soft-tissue-attenuation (S) subregions, and a 3-mm peritumoral region (R). Six prespecified feature sets (W, F, S, WF, WFS, and WFR) were modeled with L1-regularized logistic regression using nested cross-validation. Clinical and quantitative CT benchmark models were also evaluated, and the WF feature set was further compared across logistic regression, support vector machine, random forest, and XGBoost classifiers. Performance was assessed from held-out out-of-fold predictions.

**Results:**

The WF model had the highest pooled out-of-fold AUC among the six regional models (0.864; 95% CI, 0.778–0.940), but adding S or R features did not improve discrimination. The quantitative CT and clinical models achieved AUCs of 0.849 and 0.562, respectively. In a separate 10-times repeated nested cross-validation analysis, the mean fold-wise AUC difference between WF and quantitative CT was 0.023 (95% CI, −0.030 to 0.076; Holm-adjusted P = 0.389), and no pairwise comparison among the six regional models remained significant after Holm correction. When feature-set and classifier selection were repeated within the outer cross-validation loop, the selection-aware AUC was 0.842 (95% CI, 0.748–0.926). Logistic regression had the highest AUC among the four classifiers evaluated with the WF feature set. Whole-tumor mean intensity, fat-subregion run-length non-uniformity normalized, and whole-tumor GLCM correlation were the leading features in the descriptive interpretation analysis.

**Discussion:**

CT attenuation-defined regional radiomics was feasible for preoperative WDLPS–DDLPS differentiation, but the WF model did not show a significant advantage over whole-tumor radiomics or segmentation-derived quantitative CT. External validation is needed.

## 1 Introduction

Retroperitoneal sarcomas are uncommon malignancies that represent approximately 10–15% of all soft tissue sarcomas (1, 2). Because of their deep anatomical location and nonspecific early symptoms, these tumors are often detected only after they have reached a considerable size (2, 3).

Among retroperitoneal sarcomas, liposarcoma is the most common histological subtype, accounting for approximately 50–60% of cases (2, 3). Well-differentiated liposarcoma (WDLPS) generally exhibits relatively indolent growth and is characterized mainly by repeated local recurrence, whereas dedifferentiated liposarcoma (DDLPS) demonstrates more aggressive behavior, with a higher risk of distant metastasis and tumor-related mortality (3, 4). Accurate preoperative assessment of tumor differentiation is clinically important for treatment planning and risk stratification (1, 4).

In addition, retroperitoneal liposarcomas are often large tumors with complex internal architecture and heterogeneous tissue composition. Because conventional percutaneous biopsy samples only a limited portion of the lesion, it may not always reflect the overall histological composition of the tumor (5, 6). Imaging approaches that can more comprehensively characterize the spatial distribution of intratumoral components may therefore provide complementary information for clinical decision-making (1, 5).

In clinical practice, preoperative evaluation of retroperitoneal liposarcoma mainly depends on cross-sectional imaging, particularly CT and MRI (2, 7). However, differentiating WDLPS from DDLPS based on conventional imaging findings remains challenging (7–9). Imaging interpretation often provides only suggestive rather than definitive conclusions, and consistent subtype classification can be difficult to achieve. Radiologist performance has varied across studies and appears to depend on the conspicuity and distribution of non-lipomatous components within the tumor (7–9).

Although macroscopic fat and non-lipomatous soft-tissue components may be recognized on conventional CT images, their three-dimensional volumes, spatial distributions, and relative proportions are not routinely quantified in radiological reports. These measurements require segmentation-based quantitative analysis. Consequently, focal non-lipomatous or dedifferentiated areas may be obscured when a heterogeneous tumor is assessed primarily through global or qualitative imaging characteristics, thereby reducing the sensitivity of subtype identification (8, 9).

From a pathological perspective, WDLPS and DDLPS differ in the amount and spatial distribution of adipocytic and non-lipomatous tissue components (3, 7). Well-differentiated liposarcoma is predominantly composed of mature adipose tissue, whereas dedifferentiated liposarcoma usually contains variable amounts of non-lipomatous tissue with higher cellular density. These histological differences may manifest as variations in density distribution and textural patterns on CT images. Radiomics provides a method to extract a large number of quantitative imaging features from medical images and may help characterize this intratumoral heterogeneity (10–12).

However, most previous radiomics studies in retroperitoneal liposarcoma have analyzed the tumor as a whole and primarily characterized global texture heterogeneity (13, 14). In previous radiomics studies across different tumor types, subregional or habitat-based analyses have often relied on data-driven clustering or voxel-wise partitioning to identify intratumoral habitats (15). In this study, the tumor was partitioned into CT-defined fat-attenuation and soft-tissue-attenuation subregions using prespecified CT attenuation thresholds. The rationale was to examine whether diagnostically useful information was concentrated in specific tissue components instead of being captured only by whole-tumor analysis. Peritumoral imaging features may also provide complementary information related to the tumor–host interface (15). Unlike purely data-driven habitat analysis, the proposed attenuation-based approach provides an anatomically interpretable partition of the tumor. However, the resulting attenuation-defined subregions were regarded as imaging-defined surrogates rather than histologically validated tissue compartments because direct radiology–pathology correlation was not performed.

Based on these considerations, we developed a CT-based regional radiomics framework for preoperative differentiation of WDLPS and DDLPS using contrast-enhanced CT images. This study was designed as an exploratory, single-center proof-of-concept analysis. Radiomics feature sets were constructed from the whole tumor, predefined intratumoral subregions, and their combinations to investigate the contribution of different tumor components. A clinical model and a segmentation-derived quantitative CT model based on tumor diameter, tumor volume, and soft-tissue-attenuation proportion were additionally constructed as benchmark models to determine whether regional radiomics provided incremental diagnostic information. The best-performing regional feature set was subsequently evaluated using multiple machine learning classifiers. Standardized regression coefficients and descriptive SHAP analyses were used to examine the direction, relative importance, and patient-level contributions of the selected radiomics features.

## 2 Materials and methods

### 2.1 Patient cohort

This retrospective analysis was approved by the institutional review board of our institution, and the requirement for informed consent was waived. Consecutive patients who underwent surgical resection and were pathologically diagnosed with retroperitoneal liposarcoma (RLPS) at our institution between December 2013 and October 2025 were retrospectively reviewed. All included patients underwent contrast-enhanced abdominal CT within 30 days before surgery.

The inclusion criteria were as follows (1): histopathological confirmation of RLPS based on the final surgical specimen (2); availability of preoperative contrast-enhanced CT performed within 30 days before surgery; and (3) no history of other malignancies or metastatic disease. The exclusion criteria were (1): an indeterminate pathological diagnosis (2); CT image quality insufficient for segmentation or radiomics analysis; and (3) incomplete clinical data required for the analysis.

All eligible patients meeting these criteria during the study period were included. A total of 97 patients were ultimately analyzed, including 57 patients with well-differentiated liposarcoma (WDLPS) and 40 patients with dedifferentiated liposarcoma (DDLPS). Because RLPS is a rare disease and this was a retrospective study, no formal *a priori* sample-size calculation was performed. The available cohort was therefore considered an exploratory sample.

**Figure 1:**
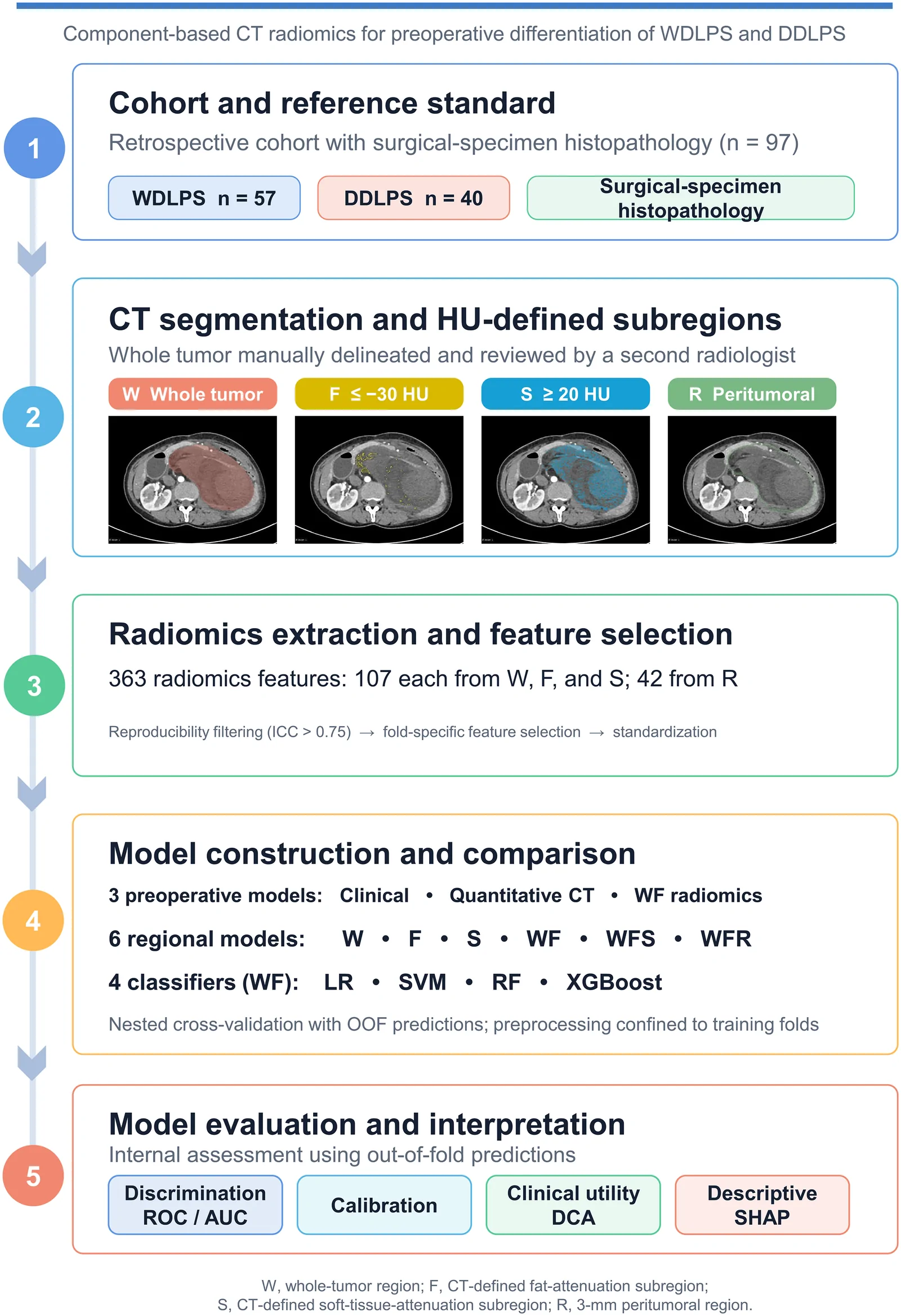
Study workflow for component-based CT radiomics analysis of WDLPS and DDLPS. The workflow comprised five stages: patient inclusion and surgical-specimen reference standard; whole-tumor segmentation and CT attenuation-based subregion definition; radiomics feature extraction and selection; construction of three preoperative models, six regional radiomics models, and four WF-based classifiers; and model evaluation using nested cross-validation. W, whole-tumor region; F, CT-defined fat-attenuation subregion; S, CT-defined soft-tissue-attenuation subregion; R, 3-mm peritumoral region; CT, computed tomography; HU, Hounsfield unit; ICC, intraclass correlation coefficient; LR, logistic regression; SVM, support vector machine; RF, random forest; XGBoost, extreme gradient boosting; OOF, out-of-fold; ROC, receiver operating characteristic; AUC, area under the ROC curve; DCA, decision curve analysis; SHAP, Shapley additive explanations; WDLPS, well-differentiated liposarcoma; DDLPS, dedifferentiated liposarcoma.

The histopathological reference standard was the final diagnosis documented in the postoperative surgical-specimen pathology report issued by the institutional pathology department. The pathological diagnoses had been established during routine clinical practice before the radiomics analysis and were therefore made without knowledge of the radiomics results. No study-specific centralized pathological rereview or repeat ancillary testing was performed. For study classification, tumors whose final pathology reports documented a dedifferentiated component were categorized as DDLPS; the remaining tumors diagnosed as WDLPS were categorized accordingly. The overall study workflow is presented in **Figure 1**.

### 2.2 CT image acquisition

Contrast-enhanced abdominal CT examinations were performed using multidetector CT scanners (Philips Healthcare or GE Healthcare). Scanning parameters included a tube voltage of 120 kV, an effective tube current ranging from 240 to 340 mA, and a reconstructed slice thickness of 1–5 mm. A nonionic iodinated contrast agent (iohexol) was administered intravenously at a dose of 1.5 mL/kg body weight with an injection rate of 3 mL/s. Arterial-phase images were acquired according to routine clinical protocols, with a scan delay of approximately 25–30 s after contrast injection.

### 2.3 Tumor segmentation and subregion definition

Three-dimensional tumor segmentation and subregion analysis were performed on preoperative contrast-enhanced CT images using 3D Slicer (version 5.8.1) (16). The whole-tumor region (W) was manually delineated slice by slice to generate a three-dimensional binary mask encompassing the entire visible tumor. Segmentation was performed by a radiologist experienced in abdominal imaging and reviewed by a senior radiologist. Disagreements were resolved by consensus. For the interobserver reproducibility analysis, a subset of cases was independently resegmented by a second radiologist.

Before attenuation-based partitioning, CT images and corresponding masks were resampled to a voxel spacing of 1 × 1 × 3 mm³. Within the W mask, voxels with attenuation values of −30 HU or lower were assigned to the CT-defined fat-attenuation subregion (F) (17), whereas voxels with values of 20 HU or higher were assigned to the CT-defined soft-tissue-attenuation subregion (S). Voxels with attenuation values between −30 and 20 HU were classified as an intermediate-attenuation component. These voxels were not analyzed as an independent regional radiomics mask but remained included in the whole-tumor region. No connected-component filtering or additional morphological processing was applied to the F or S masks.

To characterize the tumor–host interface, a three-dimensional isotropic expansion was applied to the W mask to generate a 3-mm peritumoral region (R), defined as the voxel set between the expanded mask and the original tumor boundary. Because the expanded region could extend outside the body contour or include gas-containing areas, a body mask was generated by applying a −500-HU threshold to the CT volume, retaining the largest connected component, and performing morphological closing. The R mask was then intersected with the body mask to exclude extra-body and gas-related voxels.

**Figure 2:**
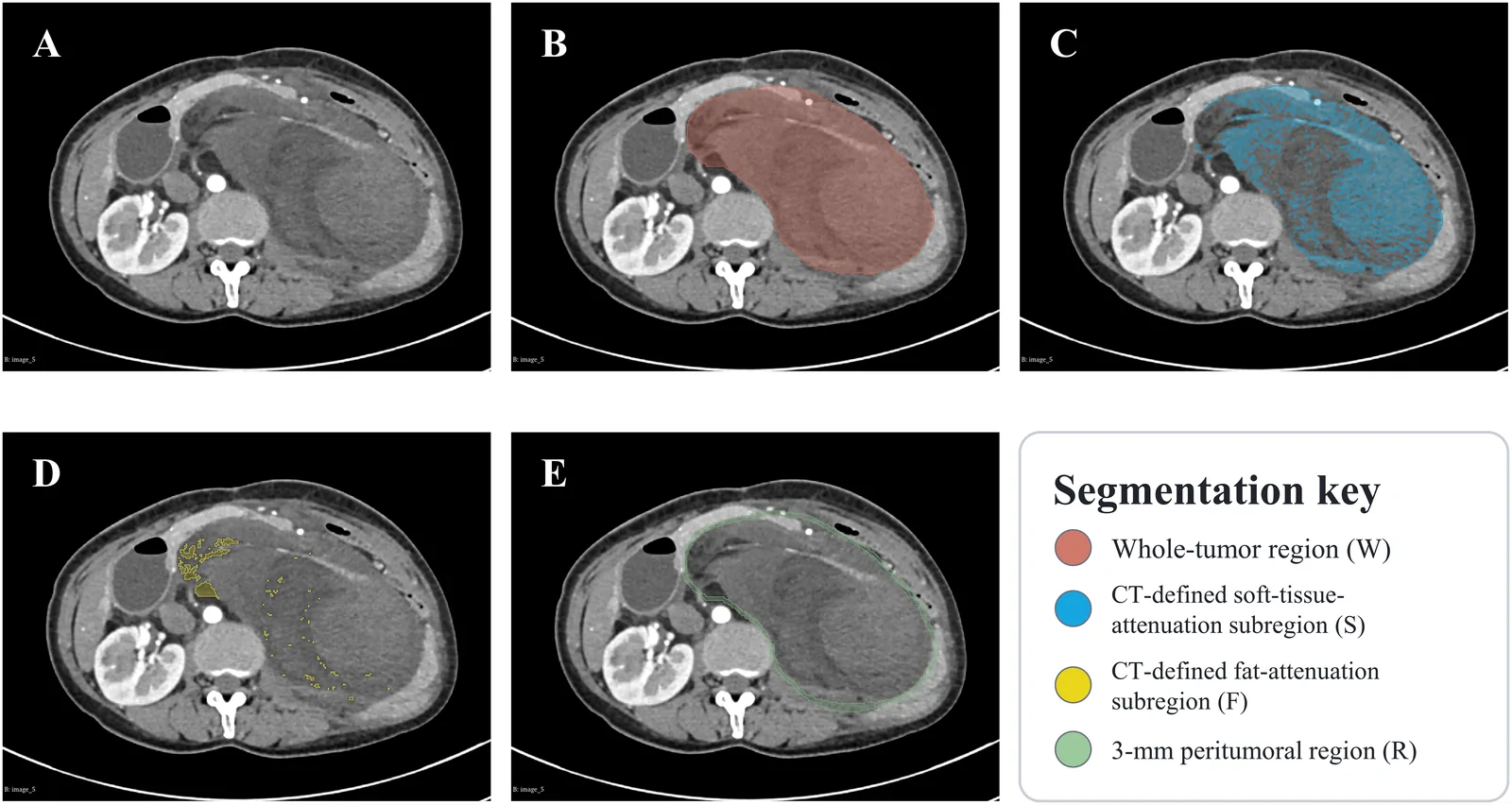
Tumor segmentation and definition of component-based subregions. **(A)** Original contrast-enhanced axial CT image. **(B)** Manually delineated whole-tumor region (W), shown in red-orange. **(C)** CT-defined soft-tissue-attenuation subregion (S), comprising voxels ≥20 HU and shown in blue. **(D)** CT-defined fat-attenuation subregion (F), comprising voxels ≤−30 HU and shown in yellow. **(E)** The 3-mm peritumoral region (R), shown in light green. Voxels between −30 and 20 HU remained within W but were not assigned to F or S. CT, computed tomography; HU, Hounsfield unit.

All W, F, S, and R masks were spatially aligned with their corresponding CT images and treated as separate volumes of interest for subsequent radiomics feature extraction. Representative examples of the segmentation and subregion definitions are shown in **Figure 2**.

### 2.4 Radiomics feature extraction

Radiomics features were extracted using PyRadiomics version 3.1.0 (12), following the Image Biomarker Standardisation Initiative recommendations (18). Images and masks were resampled to 1 × 1 × 3 mm³ in 3D Slicer, and no additional resampling was performed within PyRadiomics. This spacing standardized the in-plane resolution while limiting excessive through-plane interpolation of images reconstructed at slice thicknesses of 1–5 mm. Features were extracted from the original images without intensity normalization. A fixed bin width of 25 HU and a resegmentation range of −1000 to 3000 HU were used.

A total of 107 features were extracted separately from the W region and the F and S subregions, including shape, first-order, GLCM, GLRLM, GLSZM, GLDM, and NGTDM features. For the constructed 3-mm peritumoral region, 42 prespecified features comprising 18 first-order and 24 GLCM features were extracted; shape features were not included because the geometry of this region was determined by the expansion procedure. Thus, 363 radiomics features were initially extracted. Interobserver feature reproducibility was assessed using the independently repeated segmentations, and only features with an intraclass correlation coefficient greater than 0.75 were eligible for subsequent model development. No explicit minimum voxel count or subregion volume was prespecified. All subregions satisfied the PyRadiomics dimensionality requirement and completed feature extraction successfully.

### 2.5 Feature preprocessing and region-wise feature selection

Feature preprocessing and selection were strictly confined within the nested cross-validation framework. All preprocessing steps were fitted exclusively on the training data and subsequently applied to the corresponding held-out data to prevent information leakage.

Although no missing or infinite radiomics values were identified in the final feature matrix, a fold-specific median-imputation step was retained as a preprocessing safeguard. The imputation median was estimated exclusively from the corresponding training fold. Univariate analysis of variance (ANOVA) was then performed separately within each predefined region, and features were ranked according to their F statistics. The candidate values of k were 1, 2, 3, 5, 8, 10, 15, and 20 features per region. For combined-region models, the top k features were selected independently from each included region. The optimal value of k and the model hyperparameters were jointly determined within the inner cross-validation loop. The selected features were subsequently standardized using the mean and standard deviation estimated from the training data before model fitting.

### 2.6 Construction of regional radiomics models

Six prespecified regional radiomics feature sets were evaluated. The three single-region feature sets comprised features derived from the whole-tumor region (W), CT-defined fat-attenuation subregion (F), and CT-defined soft-tissue-attenuation subregion (S). Three combined feature sets were also evaluated: WF (W and F features), WFS (W, F, and S features), and WFR (W, F, and R features).

Each feature set was independently modeled using an L1-regularized logistic regression classifier. Model performance was evaluated using nested stratified cross-validation (19). The outer loop consisted of five folds for performance estimation, whereas the inner loop consisted of three folds for feature-number and hyperparameter optimization. Region-wise feature selection and all preprocessing steps were repeated independently within each training fold. Identical outer-fold partitions were used across the six models to facilitate paired performance comparisons.

### 2.7 Development of clinical and quantitative CT benchmark models

To evaluate the performance of radiomics relative to simpler patient and CT measurements, a clinical model and a quantitative CT model were additionally developed. The clinical model included age, sex, body mass index, and tumor side. The quantitative CT model included maximum tumor diameter, whole-tumor volume, and soft-tissue-attenuation proportion. The soft-tissue-attenuation proportion was calculated as the volume of the S subregion divided by the whole-tumor volume.

Both benchmark models were developed using L2-regularized logistic regression within the same five-fold outer and three-fold inner nested cross-validation framework. Continuous variables were standardized, whereas categorical variables were one-hot encoded. All preprocessing parameters and model hyperparameters were estimated exclusively from the corresponding training folds. Identical outer-fold partitions were used for the clinical, quantitative CT, and WF radiomics models to permit paired performance comparisons.

The clinical model, quantitative CT model, and WF radiomics model were compared in terms of discrimination, calibration, clinical utility, and threshold-dependent classification performance. Statistical comparisons of AUCs were performed using the repeated nested cross-validation procedure described in the Statistical analysis section.

### 2.8 Comparison of machine-learning classifiers and selection-aware validation

Following the comparison of the six regional radiomics models, the WF feature set, which achieved the highest numerical AUC, was carried forward for an exploratory classifier comparison. Four machine-learning classifiers—logistic regression (LR), support vector machine (SVM), random forest (RF), and extreme gradient boosting (XGBoost)—were developed using the same candidate WF feature pool.

All classifiers used the same preprocessing pipeline, region-wise feature-selection strategy, and nested cross-validation framework. Feature selection and hyperparameter optimization were performed independently for each classifier within the three-fold inner cross-validation loop, and identical five-fold outer partitions were used across classifiers. The classifier comparison was considered exploratory because selection of the regional feature set and classifier was performed using the same cohort. The classifier with the highest numerical out-of-fold AUC was carried forward for subsequent descriptive interpretability analysis.

Performance curves and classification metrics were generated from held-out out-of-fold probabilities obtained from the outer cross-validation loop. These pooled probabilities were used for descriptive assessment of discrimination, calibration, clinical utility, and classification performance, but were not used for conventional DeLong comparisons between models.

To assess potential optimism arising from sequential feature-set and classifier selection, an additional selection-aware repeated nested cross-validation analysis was performed using 10 repetitions of five-fold stratified outer cross-validation and three-fold stratified inner cross-validation. Within each outer-training dataset, the complete modeling procedure—including preprocessing, region-wise feature selection, selection among the six regional feature sets, classifier selection, hyperparameter optimization, and model fitting—was repeated without access to the corresponding outer-test fold. The resulting outer-fold predictions provided a selection-aware estimate of model performance.

### 2.9 Model interpretation

Standardized regression coefficients and odds ratios per one-standard-deviation increase were reported for the selected WF-based logistic regression model. SHAP values were subsequently calculated from the final model refitted using the entire cohort. Global feature importance was evaluated using SHAP summary and mean absolute SHAP value plots, whereas dependence and representative patient-level contribution plots were used for descriptive model interpretation. Because the final model was refitted using the complete dataset, the SHAP analysis was considered descriptive rather than independently validated.

### 2.10 Sensitivity analyses for attenuation thresholds and small subregions

To examine dependence on the selected attenuation thresholds, the fat-attenuation proportion was recalculated using thresholds of −40, −30, and −20 HU, and the soft-tissue-attenuation proportion was recalculated using thresholds of 10, 20, and 30 HU. For the fat-attenuation analysis, 1 minus the fat-attenuation proportion was used as the prediction score so that higher values indicated a greater probability of DDLPS. AUCs and 95% confidence intervals were estimated using the DeLong method, and each alternative threshold was compared with the corresponding original threshold using a paired DeLong test. These analyses evaluated the robustness of the component proportions and did not involve redevelopment of the complete radiomics models at each alternative threshold.

The volumes and proportions of the three attenuation-defined compartments were summarized for the entire cohort and separately for WDLPS and DDLPS. The presence of absent or very small subregions was also assessed. As a secondary small-region analysis, the AUC of the stored WF out-of-fold predictions was recalculated after excluding patients whose fat-attenuation subregion was smaller than 1 cm³. The WF model was not retrained in this restricted cohort, and the analysis was therefore interpreted descriptively. For this small-subregion sensitivity analysis, AUCs and their 95% confidence intervals were calculated using the DeLong method based on the stored out-of-fold predictions. These confidence intervals therefore differ slightly from the bootstrap confidence intervals reported for the primary model-performance analysis.

### 2.11 Statistical analyses

Continuous variables were summarized according to their distributions. Normally distributed variables were expressed as mean ± standard deviation and compared using the independent-samples *t* test. Non-normally distributed variables were presented as median and interquartile range and compared using the Mann–Whitney *U* test. Categorical variables were presented as counts and percentages and compared using the chi-square test or Fisher’s exact test, as appropriate.

Model discrimination was evaluated using receiver operating characteristic curves and the area under the curve. DDLPS was defined as the positive class. Accuracy, sensitivity, specificity, positive predictive value, negative predictive value, F1 score, and confusion matrices were calculated using a fixed, prespecified probability threshold of 0.50. ROC curves and AUCs were calculated from continuous predicted probabilities and were independent of this classification threshold. Unless otherwise specified, 95% confidence intervals for performance metrics were estimated using 5, 000 stratified patient-level bootstrap samples of the held-out out-of-fold predictions.

Calibration was evaluated using calibration curves, the Brier score, calibration intercept, and calibration slope. Calibration intercepts and slopes were estimated by logistic recalibration of the held-out predicted probabilities on the logit scale. Empirical calibration points were generated using five approximately equal-sized groups. Confidence intervals for the quantitative calibration measures were obtained using 5, 000 stratified patient-level bootstrap samples. Decision curve analysis was performed using held-out predicted probabilities across threshold probabilities from 0.10 to 0.90 in increments of 0.01. Pointwise 95% confidence intervals for net benefit were estimated using 5, 000 stratified patient-level bootstrap samples. Given the limited sample size, calibration and decision-curve findings were interpreted as exploratory.

Inferential comparisons between predictive models were based on the 10 repetitions of five-fold nested cross-validation using identical outer splits for the models being compared. AUC differences were calculated within the 50 matched outer-test folds. Two-sided P values and 95% confidence intervals for the mean paired AUC differences were obtained using the Nadeau–Bengio corrected resampled t test, which accounts for dependence introduced by overlapping training samples.

Holm correction was applied separately to the 15 pairwise comparisons among the six regional radiomics models and to the three prespecified comparisons among the clinical, quantitative CT, and WF radiomics models. These comparisons were considered exploratory. Conventional DeLong tests were not applied to pooled out-of-fold predictions from separately fitted models. Paired DeLong tests were used only for the alternative-HU-threshold analysis, in which fixed scalar component proportions were evaluated in the same patients.

All tests were two-sided. A P value below 0.05 was considered statistically significant before multiplicity adjustment, whereas conclusions from multiple model comparisons were based on the corresponding Holm-adjusted P values.

Statistical analyses and visualizations were performed using Python and R. Tumor segmentation was performed using 3D Slicer version 5.8.1 (16), radiomics features were extracted using PyRadiomics version 3.1.0 (12), and model development was implemented using the scikit-learn framework. The study design, analysis, and reporting were conducted with reference to the CheckList for EvaluAtion of Radiomics research recommendations (20).

## 3 Results

### 3.1 Baseline clinical and tumor characteristics

A total of 97 patients with pathologically confirmed retroperitoneal liposarcoma were included in this study, comprising 57 patients with WDLPS and 40 patients with DDLPS. All pathological diagnoses were based on postoperative surgical specimens. Baseline clinical and tumor characteristics are summarized in **Table 1**.

**Table 1:** Baseline clinical and tumor characteristics of patients with WDLPS and DDLPS.

Variables	WDLPS (n = 57)	DDLPS (n = 40)	*P* value
Age, years	57.00 (51.00–65.00)	60.00 (52.00–66.25)	0.357
BMI, kg/m²	23.31 (21.79–25.06)	23.59 (22.53–25.73)	0.391
Tumor diameter, cm	20.80 (16.10–25.60)	15.30 (11.97–19.23)	0.001
Tumor volume, cm³	1908.05 (710.01–3245.79)	1026.87 (440.01–2037.00)	0.011
Soft-tissue-attenuation proportion	0.04 (0.01–0.22)	0.78 (0.53–0.89)	<0.001
Sex			0.148
Female	31 (54.4%)	15 (37.5%)	
Male	26 (45.6%)	25 (62.5%)	
Tumor side			0.214
Left	31 (54.4%)	25 (62.5%)	
Midline	4 (7.0%)	0 (0.0%)	
Right	22 (38.6%)	15 (37.5%)	

Continuous variables are presented as median (interquartile range) unless otherwise indicated. Categorical variables are expressed as number (percentage). Comparisons between groups were performed using the independent-samples t-test or Mann–Whitney U test for continuous variables and the chi-square test or Fisher’s exact test for categorical variables, as appropriate. WDLPS, well-differentiated liposarcoma; DDLPS, dedifferentiated liposarcoma; BMI, body mass index.

No significant differences were observed between the two groups in age (57.00 [51.00–65.00] vs. 60.00 [52.00–66.25] years, *P* = 0.357), body mass index (23.31 [21.79–25.06] vs. 23.59 [22.53–25.73] kg/m², *P* = 0.391), or sex distribution (*P* = 0.148). The WDLPS group showed larger tumor diameter and tumor volume than the DDLPS group (both *P* < 0.05). In contrast, the soft-tissue-attenuation proportion was significantly higher in the DDLPS group (0.78 [0.53–0.89] vs. 0.04 [0.01–0.22], *P* < 0.001). Tumor laterality did not differ significantly between groups (*P* = 0.214). Overall, the two groups were comparable in demographic characteristics, whereas significant differences were observed in tumor size and soft-tissue-attenuation proportion.

### 3.2 Characteristics of CT attenuation-defined subregions

The distributions of the CT attenuation-defined subregions are summarized in **Supplementary Table 1**. The WDLPS group showed a higher fat-attenuation proportion than the DDLPS group (0.768 [0.242–0.952] vs. 0.010 [0.006–0.020], P < 0.001), whereas the soft-tissue-attenuation proportion was higher in the DDLPS group (0.041 [0.010–0.219] vs. 0.783 [0.527–0.890], P < 0.001). The intermediate-attenuation proportion did not differ significantly between the groups (0.120 [0.038–0.311] vs. 0.168 [0.078–0.332], P = 0.267).

Neither the fat-attenuation subregion nor the soft-tissue-attenuation subregion was absent in any patient. Six patients had a fat-attenuation subregion smaller than 1 cm³, whereas no patient had a soft-tissue-attenuation subregion smaller than 1 cm³ (**Supplementary Table 2**). All predefined regions completed radiomics feature extraction, and no missing or infinite values were identified among the 363 extracted features.

### 3.3 Performance comparison of regional radiomics models

**Figure 3:**
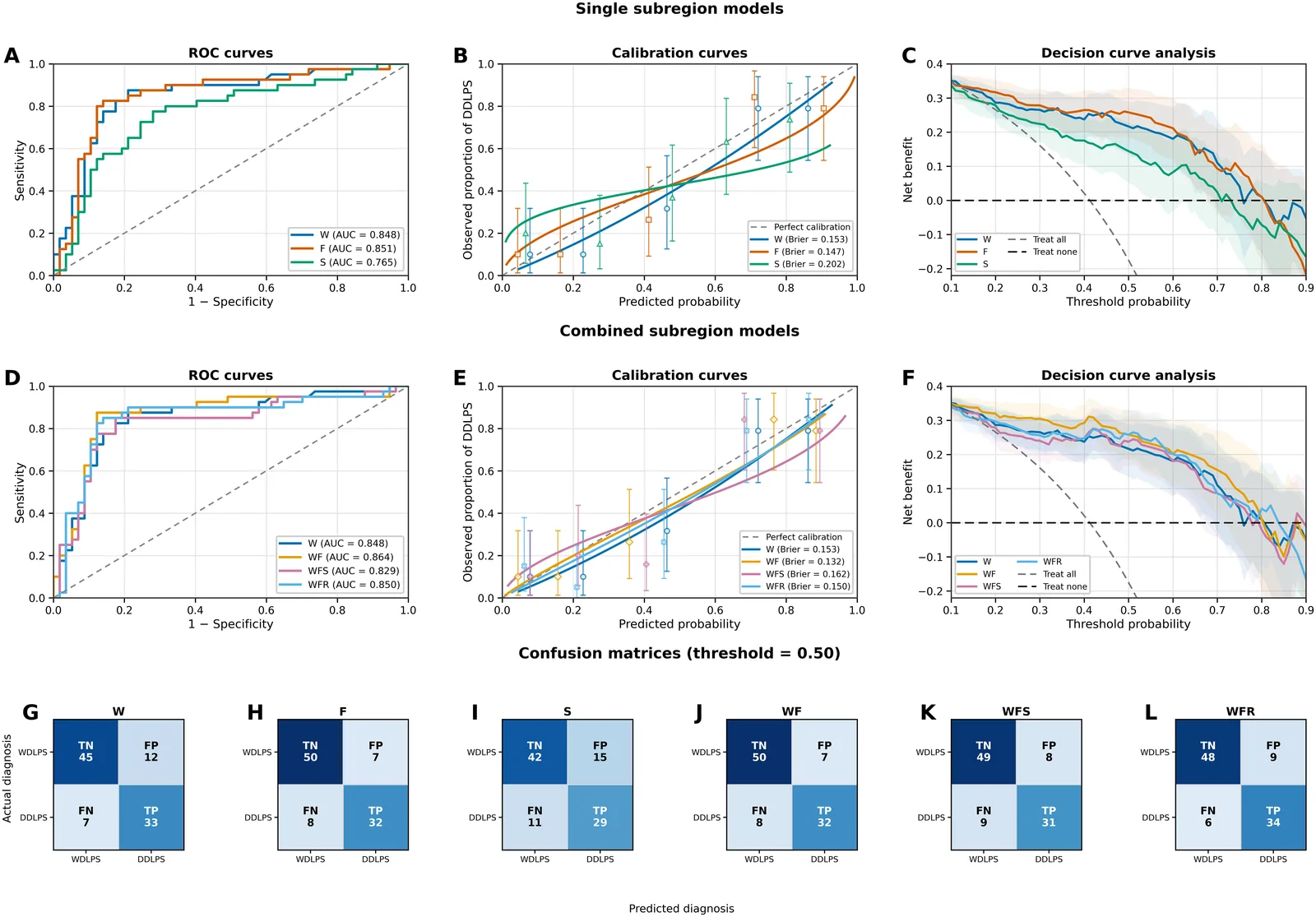
Performance of radiomics models derived from different tumor regions for differentiating WDLPS from DDLPS. **(A–C)** ROC curves, calibration curves, and DCA, respectively, for the single-region W, F, and S models. **(D–F)** Corresponding analyses for the W, WF, WFS, and WFR models. **(G–L)** Confusion matrices for W, F, S, WF, WFS, and WFR, respectively. Calibration markers represent observed proportions within probability groups, with error bars indicating 95% confidence intervals; shaded DCA areas indicate pointwise bootstrap 95% confidence intervals. Confusion matrices were calculated at a fixed probability threshold of 0.50, with DDLPS as the positive class. W, whole-tumor model; F, fat-attenuation model; S, soft-tissue-attenuation model; WF, W plus F model; WFS, W plus F and S model; WFR, W plus F and R model; R, peritumoral region; ROC, receiver operating characteristic; AUC, area under the ROC curve; DCA, decision curve analysis; TN, true negative; FP, false positive; FN, false negative; TP, true positive; WDLPS, well-differentiated liposarcoma; DDLPS, dedifferentiated liposarcoma.

Six radiomics models derived from different tumor regions and regional combinations were developed and compared (**Table 2**, **Figure 3**). Among the three single-region models, the F model achieved the highest numerical AUC (0.851), closely followed by the W model (0.848), whereas the S model showed lower discrimination (AUC = 0.765; **Figure 3A**). Among the combined-region models, the WF model achieved the highest numerical AUC of 0.864 (95% CI: 0.778–0.940). Adding soft-tissue-attenuation features in the WFS model (AUC = 0.829) or peritumoral features in the WFR model (AUC = 0.850) did not further increase the AUC relative to the WF model (**Figure 3D**).

**Table 2:** Performance of the six regional radiomics models.

Model	AUC (95% CI)	ACC (95% CI)	SEN (95% CI)	SPE (95% CI)	PPV (95% CI)	NPV (95% CI)	F1 (95% CI)
W	0.848 (0.761–0.924)	0.804 (0.722–0.876)	0.825 (0.700–0.925)	0.789 (0.684–0.895)	0.733 (0.638–0.841)	0.865 (0.786–0.941)	0.776 (0.683–0.857)
F	0.851 (0.765–0.929)	0.845 (0.773–0.918)	0.800 (0.675–0.900)	0.877 (0.789–0.947)	0.821 (0.721–0.923)	0.862 (0.789–0.932)	0.810 (0.716–0.892)
S	0.765 (0.664–0.859)	0.732 (0.639–0.814)	0.725 (0.575–0.850)	0.737 (0.614–0.842)	0.659 (0.556–0.778)	0.792 (0.706–0.880)	0.690 (0.581–0.786)
WF	0.864 (0.778–0.940)	0.845 (0.773–0.918)	0.800 (0.675–0.925)	0.877 (0.789–0.965)	0.821 (0.718–0.935)	0.862 (0.788–0.940)	0.810 (0.714–0.895)
WFS	0.829 (0.733–0.914)	0.825 (0.742–0.897)	0.775 (0.650–0.900)	0.860 (0.772–0.947)	0.795 (0.690–0.909)	0.845 (0.769–0.925)	0.785 (0.684–0.872)
WFR	0.850 (0.757–0.931)	0.845 (0.773–0.918)	0.850 (0.725–0.950)	0.842 (0.737–0.930)	0.791 (0.694–0.897)	0.889 (0.814–0.961)	0.819 (0.732–0.897)

Values are presented as estimates with 95% confidence intervals. Performance was evaluated using pooled out-of-fold predictions generated within the nested cross-validation framework. The 95% confidence intervals were estimated using 5, 000 stratified patient-level bootstrap samples. Threshold-dependent metrics were calculated using a prespecified probability threshold of 0.50, with DDLPS defined as the positive class. W, whole-tumor region; F, CT-defined fat-attenuation subregion; S, CT-defined soft-tissue-attenuation subregion; R, 3-mm peritumoral region; AUC, area under the receiver operating characteristic curve; ACC, accuracy; SEN, sensitivity; SPE, specificity; PPV, positive predictive value; NPV, negative predictive value; CI, confidence interval; DDLPS, dedifferentiated liposarcoma.

At the prespecified probability threshold of 0.50, the WF model achieved an accuracy of 0.845, sensitivity of 0.800, specificity of 0.877, and F1 score of 0.810. The corresponding confusion matrices of the six regional models are shown in **Figures 3G–L**.

In the repeated nested cross-validation comparisons, no pairwise difference among the six regional models remained statistically significant after Holm correction. Specifically, the mean paired outer-fold AUC difference between the F and S models was 0.074 (95% CI, −0.012 to 0.160; unadjusted P = 0.0897; Holm-adjusted P = 1.000). Complete pairwise comparisons are provided in **Supplementary Table 6**. Because the WF model achieved the highest numerical AUC, its candidate feature set was carried forward for the exploratory classifier comparison and subsequent descriptive model interpretation.

### 3.4 Comparison with the clinical and quantitative CT benchmark models

**Figure 4:**
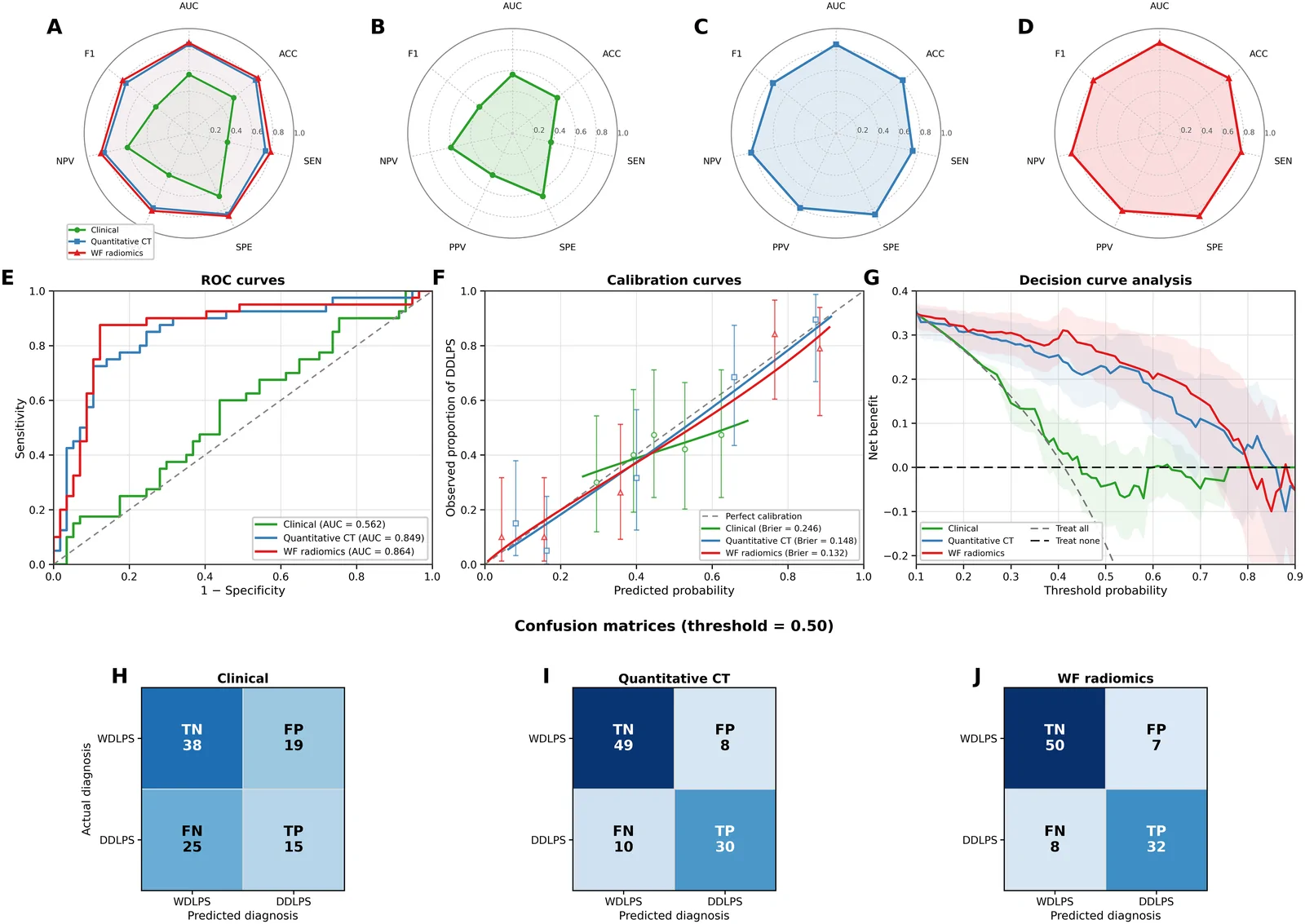
Comparison of the clinical, quantitative CT, and WF radiomics models. **(A)** Overlaid radar plot comparing the three models. **(B–D)** Individual radar plots for the clinical, quantitative CT, and WF radiomics models, respectively. All radar plots use a common scale from 0 to 1. **(E–G)** ROC curves, calibration curves, and DCA, respectively. Calibration error bars and shaded DCA areas represent 95% confidence intervals. **(H–J)** Confusion matrices for the clinical, quantitative CT, and WF radiomics models, respectively, calculated at a fixed probability threshold of 0.50 with DDLPS as the positive class. WF, whole-tumor plus fat-attenuation radiomics model; CT, computed tomography; ROC, receiver operating characteristic; DCA, decision curve analysis; AUC, area under the ROC curve; ACC, accuracy; SEN, sensitivity; SPE, specificity; PPV, positive predictive value; NPV, negative predictive value; F1, F1 score; TN, true negative; FP, false positive; FN, false negative; TP, true positive; WDLPS, well-differentiated liposarcoma; DDLPS, dedifferentiated liposarcoma.

The clinical, quantitative CT, and WF radiomics models were compared as preoperative benchmark models (**Table 3**, **Figure 4**). The clinical model showed limited discrimination, with an AUC of 0.562 (95% CI: 0.444–0.675). The quantitative CT model achieved an AUC of 0.849 (95% CI: 0.763–0.926), which was close to that of the WF radiomics model (AUC = 0.864, 95% CI: 0.778–0.940; **Figure 4E**). At the fixed probability threshold of 0.50, the corresponding accuracies were 0.546, 0.814, and 0.845, respectively. Overall performance profiles and confusion matrices are presented in **Figures 4A–D, H–J**.

**Table 3:** Performance of the clinical, quantitative CT, and WF radiomics models.

Model	AUC (95% CI)	ACC (95% CI)	SEN (95% CI)	SPE (95% CI)	PPV (95% CI)	NPV (95% CI)	F1 (95% CI)
Clinical model	0.562 (0.444–0.675)	0.546 (0.454–0.639)	0.375 (0.225–0.525)	0.667 (0.544–0.789)	0.441 (0.300–0.577)	0.603 (0.530–0.677)	0.405 (0.265–0.532)
Quantitative CT model	0.849 (0.763–0.926)	0.814 (0.742–0.887)	0.750 (0.625–0.875)	0.860 (0.772–0.947)	0.789 (0.686–0.902)	0.831 (0.758–0.909)	0.769 (0.667–0.861)
WF radiomics model	0.864 (0.778–0.940)	0.845 (0.773–0.918)	0.800 (0.675–0.925)	0.877 (0.789–0.965)	0.821 (0.718–0.935)	0.862 (0.788–0.940)	0.810 (0.714–0.895)

Values are presented as estimates with 95% confidence intervals. Performance was evaluated using pooled out-of-fold predictions generated within the nested cross-validation framework. The 95% confidence intervals were estimated using 5, 000 stratified patient-level bootstrap samples. The quantitative CT model included tumor diameter, tumor volume, and soft-tissue-attenuation proportion, whereas the clinical model included age, body mass index, sex, and tumor side. Threshold-dependent metrics were calculated using a prespecified probability threshold of 0.50, with DDLPS defined as the positive class. AUC, area under the receiver operating characteristic curve; ACC, accuracy; SEN, sensitivity; SPE, specificity; PPV, positive predictive value; NPV, negative predictive value; CI, confidence interval; CT, computed tomography; WF, radiomics model using W and F features; DDLPS, dedifferentiated liposarcoma.

In the corrected repeated nested cross-validation analysis, the mean paired outer-fold AUC difference between the WF and quantitative CT models was 0.023 (95% CI, −0.030 to 0.076; Holm-adjusted P = 0.3892). In contrast, both the WF model and quantitative CT model outperformed the clinical model after Holm correction (Holm-adjusted P = 0.000017 and 0.000088, respectively). Complete comparisons are provided in **Supplementary Table 7**.

### 3.5 Comparison of machine-learning classifiers and selection-aware validation

**Figure 5:**
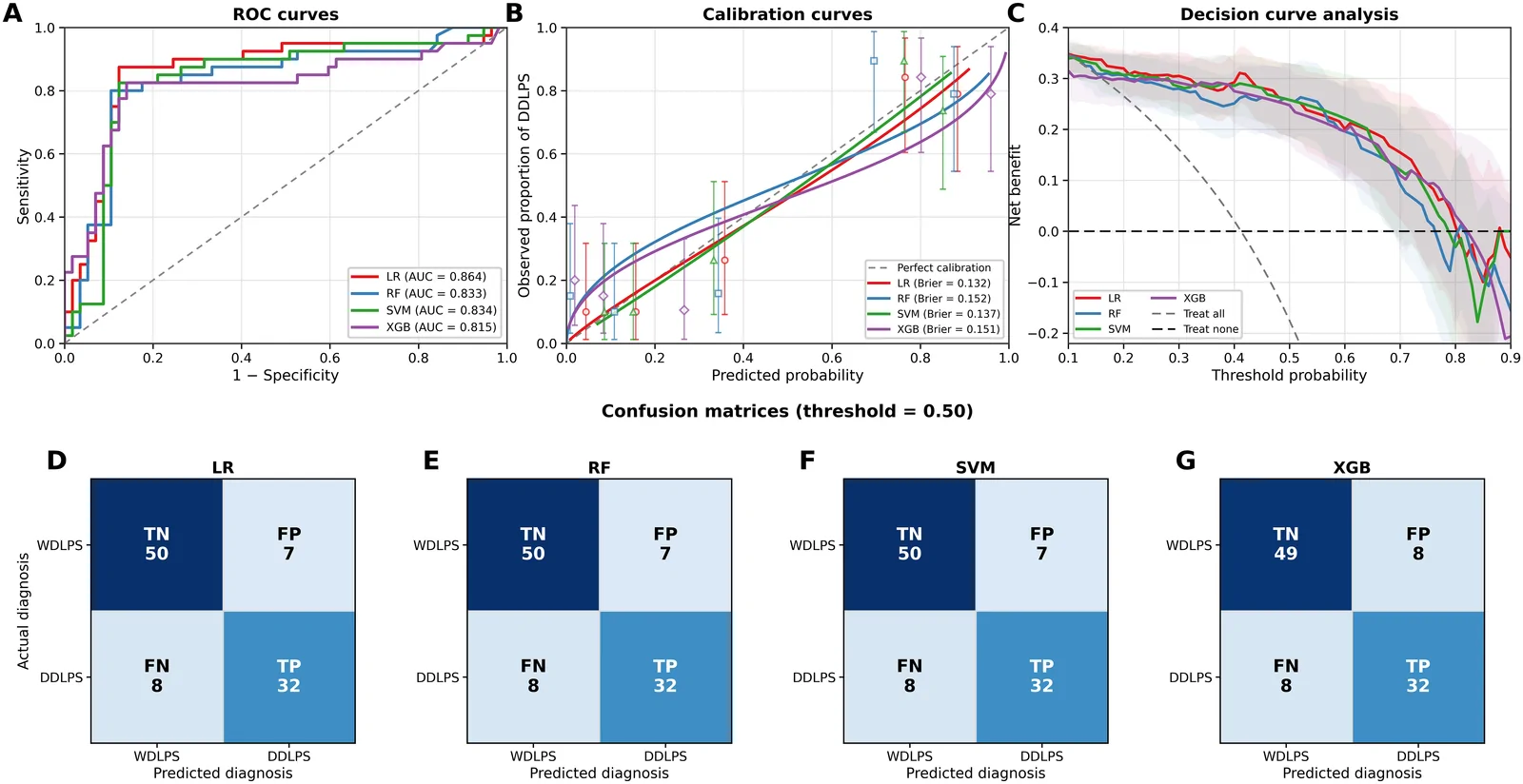
Comparison of four classifiers developed using the WF radiomics feature set. **(A–C)** ROC curves, calibration curves, and DCA, respectively, for LR, RF, SVM, and XGB. Calibration error bars and shaded DCA areas represent 95% confidence intervals. **(D–G)** Confusion matrices for LR, RF, SVM, and XGB, respectively, calculated at a fixed probability threshold of 0.50 with DDLPS as the positive class. WF, whole-tumor plus fat-attenuation feature set; LR, logistic regression; RF, random forest; SVM, support vector machine; XGB, extreme gradient boosting; ROC, receiver operating characteristic; AUC, area under the ROC curve; DCA, decision curve analysis; TN, true negative; FP, false positive; FN, false negative; TP, true positive; WDLPS, well-differentiated liposarcoma; DDLPS, dedifferentiated liposarcoma.

Following comparison of the six regional radiomics models, the WF candidate feature set, which achieved the highest numerical AUC, was carried forward for an exploratory comparison of four machine-learning classifiers: logistic regression (LR), support vector machine (SVM), random forest (RF), and extreme gradient boosting (XGBoost). ROC analysis yielded AUCs of 0.864 for LR, 0.834 for SVM, 0.833 for RF, and 0.815 for XGBoost (**Figure 5A**, **Supplementary Table 5**). Thus, LR achieved the highest numerical AUC, although the differences among the four classifiers were modest.

At the fixed probability threshold of 0.50, the LR, RF, and SVM classifiers produced identical classifications and consequently had identical confusion matrices and point estimates for the threshold-dependent performance metrics. This occurred despite differences in their continuous predicted probabilities and AUCs. The confusion matrices of the four classifiers are shown in **Figures 5D–G**.

When the complete regional feature-set and classifier selection procedure was incorporated within 10 repetitions of five-fold nested cross-validation, the selection-aware AUC was 0.842 (95% CI: 0.748–0.926). The corresponding classification metrics are provided in **Supplementary Table 5**. This estimate was slightly lower than the original numerical AUC of the WF logistic regression model but remained within a similar performance range. Across the 50 outer-training folds, WF was selected in 12 folds (24.0%) and LR in 19 folds (38.0%); however, no single feature set or classifier was selected in a majority of folds (**Supplementary Figure 1**). Based on its highest numerical AUC in the prespecified classifier comparison, the WF-based LR model was carried forward for descriptive model interpretation.

### 3.6 Calibration and decision-curve analyses

Quantitative calibration measures, including the Brier score, calibration intercept, and calibration slope, are summarized in **Supplementary Table 8**. The corresponding calibration curves are shown in **Figures 3B, E**, **4F**, **5B**. The WF-based logistic regression model had a Brier score of 0.132 (95% CI: 0.087–0.179), a calibration intercept of −0.165 (95% CI: −0.563 to 0.161), and a calibration slope of 0.885 (95% CI: 0.536–1.552). The confidence intervals for the calibration measures were relatively wide across the evaluated models.

Decision curve analyses are presented in **Figures 3C, F**, **4G**, and **5C**. Positive net benefit was observed for some models over selected threshold-probability ranges. However, the pointwise confidence intervals indicated substantial uncertainty, and no model showed consistently superior net benefit across the entire threshold range. These findings were therefore considered exploratory.

### 3.7 Sensitivity analyses

The discrimination of the attenuation-derived component proportions remained similar across the alternative HU thresholds (**Table 4**). The inverse fat-attenuation proportion yielded AUCs of 0.877, 0.883, and 0.888 at thresholds of −40, −30, and −20 HU, respectively. The soft-tissue-attenuation proportion yielded AUCs of 0.882, 0.872, and 0.868 at thresholds of 10, 20, and 30 HU, respectively. None of the alternative-threshold AUCs differed significantly from the corresponding original-threshold AUC. The distributions of component proportions at each threshold are provided in **Supplementary Table 3**.

**Table 4:** Sensitivity analysis of component proportions using alternative CT attenuation thresholds.

Component	Threshold, HU	AUC (95% CI)	ΔAUC vs baseline	Paired DeLong P
Inverse fat-attenuation proportion	−40	0.877 (0.800–0.954)	−0.006	0.294
Inverse fat-attenuation proportion	−30	0.883 (0.809–0.957)	Reference	—
Inverse fat-attenuation proportion	−20	0.888 (0.815–0.961)	0.005	0.088
Soft-tissue-attenuation proportion	10	0.882 (0.810–0.955)	0.010	0.169
Soft-tissue-attenuation proportion	20	0.872 (0.795–0.949)	Reference	—
Soft-tissue-attenuation proportion	30	0.868 (0.790–0.945)	−0.005	0.558

For the fat-attenuation analysis, 1 − fat-attenuation proportion was used as the prediction score so that higher values indicated a greater probability of DDLPS. The prespecified baseline thresholds were −30 HU for the fat-attenuation subregion and 20 HU for the soft-tissue-attenuation subregion. AUCs and 95% confidence intervals were estimated using the DeLong method. AUCs obtained using alternative thresholds were compared with those obtained using the corresponding baseline threshold using paired DeLong tests. The reported P values are unadjusted, and these sensitivity analyses were considered exploratory. Abbreviations: AUC, area under the receiver operating characteristic curve; CI, confidence interval; CT, computed tomography; DDLPS, dedifferentiated liposarcoma; HU, Hounsfield unit.

After excluding the six patients with a fat-attenuation subregion smaller than 1 cm³, the AUC of the stored WF out-of-fold predictions was 0.883 (DeLong 95% CI, 0.799–0.966), compared with 0.864 (DeLong 95% CI, 0.780–0.947) in the complete cohort (**Supplementary Table 4**). Because the model was not retrained and the two estimates were obtained from different patient subsets, this analysis was interpreted descriptively.

### 3.8 Model interpretation

**Figure 6:**
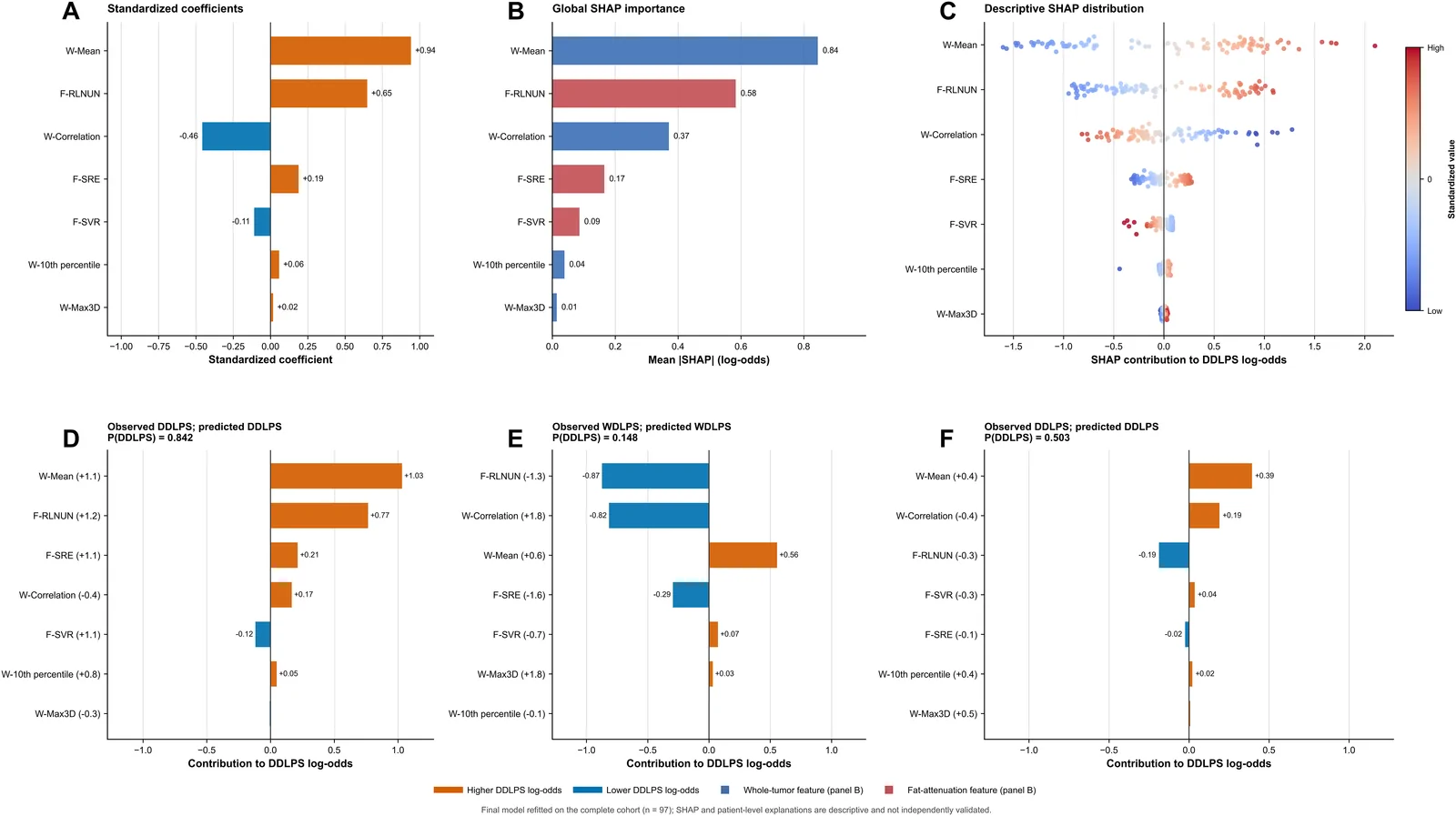
Coefficient- and SHAP-based interpretation of the final WF logistic regression model. **(A)** Standardized regression coefficients of the seven retained features. **(B)** Global feature importance ranked by the mean absolute SHAP value. **(C)** Distribution of SHAP values across the cohort, colored by standardized feature value. **(D–F)** Feature contributions for three representative patients. Orange and blue indicate contributions toward higher and lower DDLPS log-odds, respectively; in panel B, blue and red denote whole-tumor and fat-attenuation features. SHAP values are reported on the log-odds scale. The analysis was based on the final model refitted to all 97 patients and is descriptive rather than independently validated. W, whole-tumor feature; F, fat-attenuation feature; RLNUN, run-length nonuniformity normalized; SRE, short-run emphasis; SVR, surface-to-volume ratio; Max3D, maximum three-dimensional diameter; WF, whole-tumor plus fat-attenuation feature set; SHAP, Shapley additive explanations; WDLPS, well-differentiated liposarcoma; DDLPS, dedifferentiated liposarcoma.

The final WF-based logistic regression model retained seven radiomics features (**Figure 6A**). Whole-tumor mean intensity (W-Mean) had the largest positive standardized coefficient (β = 0.942; OR per 1-SD increase = 2.564), followed by run-length non-uniformity normalized from the fat-attenuation subregion (F-RLNUN; β = 0.648; OR = 1.912). Whole-tumor GLCM correlation (W-Correlation) showed a negative coefficient (β = −0.457; OR = 0.633). The complete standardized coefficients and corresponding odds ratios are provided in **Supplementary Table 9**.

The descriptive SHAP analysis produced a similar feature-importance ranking (**Figures 6B, C**), with W-Mean, F-RLNUN, and W-Correlation identified as the three leading features. Of these, F-RLNUN originated from the CT-defined fat-attenuation subregion, whereas W-Mean and W-Correlation were whole-tumor features. Representative patient-level contribution plots illustrate how the selected features contributed to individual model outputs (**Figures 6D–F**). Because the interpretation analysis was based on the final model refitted using the complete cohort, these findings were considered descriptive rather than independently validated.

## 4 Discussion

In this 97-patient cohort, the WF model yielded the highest AUC among the six regional radiomics models, but the improvement over whole-tumor radiomics was small and not statistically significant. A quantitative CT model based on tumor diameter, volume, and soft-tissue-attenuation proportion performed similarly. These results suggest that tumor size and attenuation-defined tissue composition capture a substantial part of the imaging information associated with WDLPS–DDLPS differentiation, whereas the additional contribution of regional texture features appears limited in the present cohort.

The quantitative CT model performed similarly to the WF model (AUC, 0.849 vs. 0.864). It included maximum tumor diameter, whole-tumor volume, and soft-tissue-attenuation proportion—measurements that are less complex than radiomics features but still require three-dimensional tumor segmentation and are not routinely reported in clinical practice. The close performance of the two models indicates that tumor size and the soft-tissue-attenuation proportion captured much of the information relevant to subtype discrimination. No statistically significant incremental value was observed for WF over these segmentation-derived measurements.

Previous radiomics studies of retroperitoneal liposarcoma have generally relied on whole-tumor features, whereas subregional studies in other tumor types often define intratumoral habitats through data-driven clustering. We instead applied prespecified HU thresholds to define fat-attenuation and soft-tissue-attenuation subregions. This rule-based partition was designed as an interpretable imaging construct rather than a histopathological classification. The relatively high AUCs of the F and WF models indicate that variation within the fat-attenuation subregion may contribute to subtype discrimination. Such variation could arise from differences in adipose architecture, attenuation heterogeneity, or partial-volume effects, although these possibilities cannot be distinguished without spatial radiology–pathology correlation. Because WF was not significantly better than the whole-tumor model, F features should be regarded as complementary to, rather than superior to, whole-tumor features.

The −30- and 20-HU thresholds were operational imaging cutoffs rather than histological boundaries. The −30-HU cutoff was based on previous CT fat-segmentation studies (17), whereas the 20-HU cutoff was prespecified to identify clearly nonadipose voxels. Voxels between these thresholds remained part of the whole-tumor region but were not analyzed as a separate radiomics subregion. Changing the thresholds produced little variation in discrimination: AUCs ranged from 0.877 to 0.888 for the inverse fat-attenuation proportion and from 0.868 to 0.882 for the soft-tissue-attenuation proportion, with no significant differences from the original thresholds. This sensitivity analysis assessed the attenuation-derived proportions only; the complete radiomics pipeline was not repeated at each threshold.

Notably, the attenuation-derived component proportions themselves showed substantial discriminatory ability. Because these scalar measurements did not require model fitting, their AUCs were calculated directly in the complete cohort and should not be directly compared with the cross-validated AUCs of the radiomics models. Nevertheless, their performance suggests that differences in tissue composition account for a substantial proportion of the CT information associated with WDLPS–DDLPS differentiation. The potential incremental contribution of radiomics texture features beyond these simpler quantitative measurements therefore warrants further evaluation in independent cohorts.

Both subregions were present in every patient, although six patients had a fat-attenuation subregion smaller than 1 cm³. After these patients were excluded, the AUC of the stored WF out-of-fold predictions was 0.883, compared with 0.864 in the full cohort. Because the model was not retrained and the estimates were obtained from different patient sets, this result should be regarded only as a sensitivity check. CT-based fat quantification is sensitive to contrast enhancement and acquisition parameters (21). Attenuation-based partitioning may also vary with reconstruction settings, image noise, interpolation, and partial-volume effects. Its reproducibility therefore requires evaluation using standardized acquisition protocols.

The peritumoral analysis also needs to be interpreted cautiously. The WFR model yielded an AUC of 0.850, similar to that of the whole-tumor model (0.848) and below the observed AUC of the WF model (0.864). These differences were not statistically significant after Holm correction. A fixed 3-mm expansion in the retroperitoneum can include adjacent fat, bowel, muscle, and vascular structures, potentially diluting features specific to the tumor–host interface. Peritumoral definitions also differ across studies in expansion width and the treatment of surrounding tissues. The absence of incremental discrimination in this cohort therefore applies to the present 3-mm definition and does not rule out a contribution from the tumor–host interface.

Using the WF feature set, logistic regression yielded an AUC of 0.864, compared with 0.834 for support vector machine, 0.833 for random forest, and 0.815 for XGBoost. At the fixed threshold of 0.50, logistic regression, support vector machine, and random forest assigned every patient to the same class despite differences in their continuous probability estimates. When feature-set and classifier selection were repeated within nested cross-validation, the AUC was 0.842; WF was selected in 12 of 50 outer-training folds and logistic regression in 19 of 50 folds. The absence of a dominant feature set or classifier indicates that several candidate pipelines performed similarly. More complex classifiers offered no clear advantage in this cohort, possibly because only 97 patients and a small number of retained features were available. This result does not establish that logistic regression would remain preferable in a larger external cohort.

The standardized regression coefficients provided the primary feature-level interpretation of the final WF-based logistic regression model. W-Mean and F-RLNUN showed the largest positive coefficients, whereas W-Correlation showed a negative coefficient. The descriptive SHAP analysis produced a similar importance ranking. Among these three leading features, only F-RLNUN originated from the CT-defined fat-attenuation subregion, whereas W-Mean and W-Correlation were whole-tumor features. Thus, the final model incorporated information from both the whole-tumor and fat-attenuation feature sets rather than relying predominantly on one subregion. Because SHAP values were calculated from the final model refitted using the complete cohort, these explanations were descriptive and were not independently validated. Moreover, the approximately linear SHAP patterns were expected for a logistic regression model and should not be interpreted as evidence of complex biological relationships. Direct radiology–pathology correlation is required to clarify the biological relevance of these imaging features.

Several limitations of this study should be acknowledged. The retrospective, single-center cohort comprised 97 patients, and no formal *a priori* sample-size calculation was performed. Although the complete feature-set and classifier selection procedure was repeated within nested cross-validation, model development and comparison were still confined to the same cohort. In the absence of an external or temporal validation cohort, residual optimism in the performance estimates remains possible, and the generalizability of the models is unknown.

The CT examinations spanned nearly 12 years and were acquired using different scanners and routine clinical protocols. Resampling standardized voxel spacing but did not eliminate variation in scanner generation, reconstruction kernel, slice thickness, contrast timing, image noise, or partial-volume effects. Because detailed acquisition metadata were unavailable for some patients, performance could not be examined reliably across scanners, acquisition periods, or reconstruction settings. Histopathological diagnoses were obtained from routine postoperative surgical-specimen reports without a study-specific centralized rereview. The WHO classification edition and the use of MDM2 or CDK4 ancillary testing were also not consistently documented.

The fat-attenuation and soft-tissue-attenuation subregions were operational imaging surrogates and were not validated by direct spatial radiology–pathology correlation. The alternative-threshold analysis assessed the discrimination of attenuation-derived proportions but did not repeat the complete radiomics pipeline at each threshold. No minimum subregion volume was prespecified, and six patients had fat-attenuation subregions smaller than 1 cm³. The analysis based on their exclusion used stored predictions and did not directly evaluate the stability of texture features extracted from small masks. Manual tumor delineation remained another source of variability despite ICC-based feature screening. In addition, the calibration and decision-curve estimates had wide confidence intervals, and the SHAP analysis described a model refitted to the full cohort. These limitations restrict conclusions about calibration, clinical utility, and the biological meaning of the selected features.

Overall, this exploratory single-center study supports the feasibility of extracting radiomics features from CT attenuation-defined tumor subregions for differentiating WDLPS from DDLPS. Although the WF model achieved the highest numerical AUC, it did not significantly outperform either the whole-tumor radiomics model or the segmentation-derived quantitative CT model. The present findings therefore do not establish the clinical applicability or superiority of component-based radiomics. External validation in larger multicenter cohorts, preferably using standardized CT acquisition protocols and direct radiology–pathology correlation, is required.

## 5 Conclusion

CT attenuation-defined regional radiomics was feasible for preoperative differentiation of WDLPS and DDLPS. The WF model yielded the highest AUC, but its performance was not significantly better than whole-tumor radiomics or a simpler quantitative CT model. These findings suggest that attenuation-defined subregions may complement whole-tumor analysis, although their incremental value remains uncertain. Multicenter external validation is needed before clinical application.

## Data Availability

The raw data supporting the conclusions of this article will be made available by the authors, without undue reservation.
