# Supplementary material for: Component-based CT radiomics for preoperative differentiation of well-differentiated and dedifferentiated retroperitoneal liposarcoma
Source: Front Oncol. 2026 Sep 9;16:1924254. doi: 10.3389/fonc.2026.1924254 (PMC13597326; doi:10.3389/fonc.2026.1924254)
Supplement: Supplementary file 1 [file SupplementaryFile1.docx]

**Supplementary Figure S1. Detailed workflow of the component-based radiomics model development.** The diagram illustrates the complete modeling pipeline, including tumor segmentation, radiomics feature extraction, feature preprocessing, nested cross-validation, regional feature-set comparison, classifier evaluation, and final SHAP interpretation.

**Supplementary Table S1.** Performance metrics of different machine learning classifiers developed using the WF feature set

| **Model** | **AUC** | **Accuracy** | **Sensitivity** | **Specificity** | **PPV** | **NPV** | **F1** |
| --- | --- | --- | --- | --- | --- | --- | --- |
| LR | 0.864 | 0.845 | 0.800 | 0.877 | 0.820 | 0.862 | 0.810 |
| RF | 0.832 | 0.845 | 0.800 | 0.877 | 0.820 | 0.862 | 0.810 |
| SVM | 0.833 | 0.845 | 0.800 | 0.877 | 0.820 | 0.862 | 0.810 |
| XGB | 0.815 | 0.835 | 0.800 | 0.859 | 0.800 | 0.859 | 0.800 |
