# Supplementary material for: Component-based CT radiomics for preoperative differentiation of well-differentiated and dedifferentiated retroperitoneal liposarcoma
Source: Front Oncol. 2026 Sep 9;16:1924254. doi: 10.3389/fonc.2026.1924254 (PMC13597326; doi:10.3389/fonc.2026.1924254)
Supplement: Supplementary file 2 [file SupplementaryFile2.docx]

**Supplementary Methods: Audit and sensitivity analysis of CT attenuation-defined subregions**

Within the whole-tumor mask, voxels with attenuation values of −30 HU or lower were assigned to the CT-defined fat-attenuation subregion, whereas voxels with attenuation values of 20 HU or higher were assigned to the CT-defined soft-tissue-attenuation subregion. Voxels between −30 and 20 HU were classified as the intermediate-attenuation compartment. Although the intermediate-attenuation compartment was not analyzed as an independent radiomics subregion, these voxels remained included in the whole-tumor region. The volumes and proportions of the three attenuation-defined compartments were calculated for each patient.

The presence of absent or very small fat-attenuation and soft-tissue-attenuation subregions was assessed. No explicit minimum voxel count or volume was prespecified for radiomics extraction. PyRadiomics required a minimum region-of-interest dimensionality of two, and both attenuation-defined subregion masks satisfied this requirement.

To evaluate the sensitivity of the results to the selected HU thresholds, the fat-attenuation proportion was recalculated using thresholds of −40, −30, and −20 HU, and the soft-tissue-attenuation proportion was recalculated using thresholds of 10, 20, and 30 HU. ROC AUCs and 95% confidence intervals were estimated using the DeLong method. AUCs obtained at alternative thresholds were compared with those obtained at the original thresholds using paired DeLong tests. In an additional small-region sensitivity analysis, the AUC of the original WF out-of-fold predictions was recalculated after excluding patients with fat-attenuation subregions smaller than 1 cm³.

**Supplementary Table S1. Distributions of CT attenuation-defined tumor subregions in patients with WDLPS and DDLPS**

| Variable | WDLPS | DDLPS | Overall | P value |
| --- | --- | --- | --- | --- |
| Fat-attenuation volume, cm³ | 1094.65 (293.79–2575.19) | 8.92 (4.20–37.87) | 226.11 (8.81–1581.21) | <0.001 |
| Soft-tissue-attenuation volume, cm³ | 79.02 (27.19–331.17) | 613.20 (270.09–1265.16) | 197.79 (40.83–710.50) | <0.001 |
| Intermediate-attenuation volume, cm³ | 169.75 (34.94–1189.45) | 143.59 (49.00–455.96) | 149.60 (36.32–535.91) | 0.545 |
| Fat-attenuation proportion | 0.768 (0.242–0.952) | 0.010 (0.006–0.020) | 0.234 (0.010–0.894) | <0.001 |
| Soft-tissue-attenuation proportion | 0.041 (0.010–0.219) | 0.783 (0.527–0.890) | 0.242 (0.022–0.801) | <0.001 |
| Intermediate-attenuation proportion | 0.120 (0.038–0.311) | 0.168 (0.078–0.332) | 0.147 (0.054–0.311) | 0.267 |

Values are presented as median (interquartile range). The CT-defined fat-attenuation subregion comprised whole-tumor voxels with attenuation values ≤−30 HU, whereas the CT-defined soft-tissue-attenuation subregion comprised voxels with attenuation values ≥20 HU. Voxels between −30 and 20 HU were classified as the intermediate-attenuation compartment. P values were calculated using the Mann–Whitney U test. Abbreviations: CT, computed tomography; WDLPS, well-differentiated liposarcoma; DDLPS, dedifferentiated liposarcoma; HU, Hounsfield unit.

**Supplementary Table S2. Frequency of absent or small attenuation-defined subregions**

| Region | Cohort | N | Absent, n | <64 voxels, n | <100 voxels, n | <1 cm³, n | Failed dimensionality, n | Minimum voxel count | Minimum volume, cm³ |
| --- | --- | --- | --- | --- | --- | --- | --- | --- | --- |
| Fat-attenuation subregion | WDLPS | 57 | 0 | 1 | 1 | 2 | 0 | 42 | 0.126 |
| Fat-attenuation subregion | DDLPS | 40 | 0 | 1 | 2 | 4 | 0 | 16 | 0.048 |
| Fat-attenuation subregion | Overall | 97 | 0 | 2 | 3 | 6 | 0 | 16 | 0.048 |
| Soft-tissue-attenuation subregion | WDLPS | 57 | 0 | 0 | 0 | 0 | 0 | 426 | 1.278 |
| Soft-tissue-attenuation subregion | DDLPS | 40 | 0 | 0 | 0 | 0 | 0 | 2782 | 8.346 |
| Soft-tissue-attenuation subregion | Overall | 97 | 0 | 0 | 0 | 0 | 0 | 426 | 1.278 |

The resampled CT images and masks used for radiomics extraction had a voxel spacing of 1 × 1 × 3 mm, corresponding to a voxel volume of 0.003 cm³. An absent subregion was defined as a mask containing no voxels. No explicit minimum voxel count or volume was prespecified before radiomics extraction (minimumROISize=None). PyRadiomics required a minimum region-of-interest dimensionality of two (minimumROIDimensions=2), and all masks for the two attenuation-defined subregions satisfied this requirement. Abbreviations: CT, computed tomography; WDLPS, well-differentiated liposarcoma; DDLPS, dedifferentiated liposarcoma.

**Supplementary Table S3. Distributions of attenuation-derived proportions using alternative HU thresholds**

| Attenuation-derived variable | Threshold, HU | WDLPS | DDLPS | Overall |
| --- | --- | --- | --- | --- |
| Fat-attenuation proportion | -40 | 0.728 (0.162–0.924) | 0.006 (0.004–0.016) | 0.137 (0.006–0.844) |
| Fat-attenuation proportion | -30 | 0.768 (0.242–0.952) | 0.010 (0.006–0.020) | 0.234 (0.010–0.894) |
| Fat-attenuation proportion | -20 | 0.808 (0.331–0.959) | 0.017 (0.010–0.036) | 0.275 (0.017–0.906) |
| Soft-tissue-attenuation proportion | 10 | 0.073 (0.015–0.352) | 0.907 (0.747–0.949) | 0.414 (0.037–0.918) |
| Soft-tissue-attenuation proportion | 20 | 0.041 (0.010–0.219) | 0.783 (0.527–0.890) | 0.242 (0.022–0.801) |
| Soft-tissue-attenuation proportion | 30 | 0.020 (0.007–0.121) | 0.575 (0.246–0.761) | 0.133 (0.012–0.580) |

Values are presented as median (interquartile range). The fat-attenuation proportion was calculated using thresholds of −40, −30, and −20 HU. The soft-tissue-attenuation proportion was calculated using thresholds of 10, 20, and 30 HU. The original thresholds were −30 HU for the fat-attenuation subregion and 20 HU for the soft-tissue-attenuation subregion. Abbreviations: WDLPS, well-differentiated liposarcoma; DDLPS, dedifferentiated liposarcoma; HU, Hounsfield unit.

**Supplementary Table S4. Sensitivity analysis of the original WF model after excluding very small fat-attenuation subregions**

| Analysis | N | WDLPS | DDLPS | AUC (95% CI) |
| --- | --- | --- | --- | --- |
| Original WF OOF predictions, all patients | 97 | 57 | 40 | 0.864 (0.780–0.947) |
| Original WF OOF predictions, excluding F <1 cm³ | 91 | 55 | 36 | 0.883 (0.799–0.966) |

Very small fat-attenuation subregions were defined as those with a volume <1 cm³ at the original threshold of −30 HU. The analysis was based on the original stored out-of-fold predictions of the WF model. The model was not retrained after patient exclusion. AUCs and 95% confidence intervals were estimated using the DeLong method. Because the full-cohort and restricted-cohort AUCs were calculated in different patient samples, no formal paired statistical comparison was performed. Abbreviations: WF, model using W and F features; AUC, area under the receiver operating characteristic curve; CI, confidence interval; HU, Hounsfield unit.

**Supplementary Table S5. Diagnostic performance of the selection-aware repeated nested cross-validation procedure and prespecified radiomics models for differentiating well-differentiated from dedifferentiated retroperitoneal liposarcoma.**

| Model | AUC | Accuracy | Sensitivity | Specificity | PPV | NPV | F1 |
| --- | --- | --- | --- | --- | --- | --- | --- |
| Entire regional feature-set and classifier selection procedure | 0.842 (0.748-0.926) | 0.845 (0.773-0.918) | 0.800 (0.675-0.925) | 0.877 (0.789-0.947) | 0.821 (0.721-0.921) | 0.862 (0.791-0.941) | 0.810 (0.718-0.894) |
| W | 0.848 (0.761-0.924) | 0.804 (0.722-0.876) | 0.825 (0.700-0.925) | 0.789 (0.684-0.895) | 0.733 (0.638-0.841) | 0.865 (0.786-0.941) | 0.776 (0.683-0.857) |
| F | 0.851 (0.765-0.929) | 0.845 (0.773-0.918) | 0.800 (0.675-0.900) | 0.877 (0.789-0.947) | 0.821 (0.721-0.923) | 0.862 (0.789-0.932) | 0.810 (0.716-0.892) |
| S | 0.765 (0.664-0.859) | 0.732 (0.639-0.814) | 0.725 (0.575-0.850) | 0.737 (0.614-0.842) | 0.659 (0.556-0.778) | 0.792 (0.706-0.880) | 0.690 (0.581-0.786) |
| WF | 0.864 (0.778-0.940) | 0.845 (0.773-0.918) | 0.800 (0.675-0.925) | 0.877 (0.789-0.965) | 0.821 (0.718-0.935) | 0.862 (0.788-0.940) | 0.810 (0.714-0.895) |
| WFS | 0.829 (0.733-0.914) | 0.825 (0.742-0.897) | 0.775 (0.650-0.900) | 0.860 (0.772-0.947) | 0.795 (0.690-0.909) | 0.845 (0.769-0.925) | 0.785 (0.684-0.872) |
| WFR | 0.850 (0.757-0.931) | 0.845 (0.773-0.918) | 0.850 (0.725-0.950) | 0.842 (0.737-0.930) | 0.791 (0.694-0.897) | 0.889 (0.814-0.961) | 0.819 (0.732-0.897) |
| RF | 0.833 (0.742-0.915) | 0.845 (0.773-0.907) | 0.800 (0.675-0.925) | 0.877 (0.789-0.947) | 0.821 (0.720-0.921) | 0.862 (0.787-0.939) | 0.810 (0.712-0.894) |
| SVM | 0.834 (0.741-0.918) | 0.845 (0.773-0.918) | 0.800 (0.675-0.925) | 0.877 (0.789-0.947) | 0.821 (0.721-0.923) | 0.862 (0.787-0.938) | 0.810 (0.712-0.894) |
| XGB | 0.815 (0.715-0.905) | 0.835 (0.763-0.907) | 0.800 (0.675-0.925) | 0.860 (0.772-0.947) | 0.800 (0.702-0.909) | 0.860 (0.785-0.933) | 0.800 (0.703-0.886) |

Values are presented as estimates with 95% confidence intervals. The first row reports the performance of the complete selection-aware procedure, in which regional feature-set selection, classifier selection, feature selection, and hyperparameter optimization were performed exclusively within the inner cross-validation loop. Repeated nested cross-validation comprised 10 repetitions of five-fold outer cross-validation with three-fold inner cross-validation. For each patient, out-of-fold predicted probabilities were averaged across the 10 repetitions before performance evaluation. W, F, S, WF, WFS, and WFR represent the six prespecified regional models constructed using logistic regression. RF, SVM, and XGB represent alternative classifiers constructed using the WF feature set; the WF row therefore corresponds to the WF logistic regression model. All performance estimates were derived from out-of-fold predictions. The 95% confidence intervals were calculated using 5,000 patient-level stratified bootstrap resamples. DDLPS was defined as the positive class. A fixed probability threshold of 0.50 was used to calculate accuracy, sensitivity, specificity, PPV, NPV, and F1 score, whereas AUC was calculated from continuous predicted probabilities. Abbreviations: W, whole-tumor region; F, CT-defined fat-attenuation subregion; S, CT-defined soft-tissue-attenuation subregion; R, 3-mm peritumoral region; WF, model using W and F features; WFS, model using W, F, and S features; WFR, model using W, F, and R features; LR, logistic regression; RF, random forest; SVM, support vector machine; XGB, extreme gradient boosting; AUC, area under the receiver operating characteristic curve; PPV, positive predictive value; NPV, negative predictive value; WDLPS, well-differentiated liposarcoma; DDLPS, dedifferentiated liposarcoma; CI, confidence interval.

**Supplementary Table S6.** **Exploratory pairwise comparisons among the six regional radiomics models using corrected repeated nested cross-validation.**

| Comparison | Delta AUC (95% CI) | Unadjusted P value | Holm-adjusted P value |
| --- | --- | --- | --- |
| W vs F | 0.006 (-0.049 to 0.061) | 0.824 | 1 |
| W vs S | 0.080 (0.006 to 0.154) | 0.0356 | 0.5333 |
| W vs WF | 0.006 (-0.038 to 0.050) | 0.7821 | 1 |
| W vs WFS | 0.023 (-0.020 to 0.066) | 0.2855 | 1 |
| W vs WFR | 0.010 (-0.036 to 0.057) | 0.6544 | 1 |
| F vs S | 0.074 (-0.012 to 0.160) | 0.0897 | 1 |
| F vs WF | -0.000 (-0.040 to 0.040) | 0.9992 | 1 |
| F vs WFS | 0.017 (-0.031 to 0.066) | 0.4833 | 1 |
| F vs WFR | 0.004 (-0.050 to 0.058) | 0.8743 | 1 |
| S vs WF | -0.074 (-0.150 to 0.003) | 0.058 | 0.8114 |
| S vs WFS | -0.057 (-0.122 to 0.008) | 0.0847 | 1 |
| S vs WFR | -0.070 (-0.147 to 0.007) | 0.0756 | 0.9832 |
| WF vs WFS | 0.017 (-0.025 to 0.059) | 0.4218 | 1 |
| WF vs WFR | 0.004 (-0.040 to 0.049) | 0.8473 | 1 |
| WFS vs WFR | -0.013 (-0.060 to 0.035) | 0.5918 | 1 |

Values are based on 50 matched outer-test folds from 10 repetitions of five-fold nested cross-validation. Within each outer-training set, preprocessing, region-wise feature selection, hyperparameter optimization, and model fitting were repeated using three-fold inner cross-validation. ΔAUC was calculated as the mean outer-fold AUC of the first model minus that of the second model and may therefore differ from the difference between the aggregate AUCs reported in Table 2. The unadjusted 95% confidence intervals and two-sided P values were obtained using the Nadeau–Bengio corrected resampled *t*-test. Holm correction was applied to the 15 pairwise comparisons. Positive ΔAUC values favor the first model. All comparisons were exploratory.

**Supplementary Table S7. Exploratory pairwise comparisons among the clinical, quantitative CT, and WF radiomics models.**

| Comparison | Delta AUC (95% CI) | Unadjusted P value | Holm-adjusted P value |
| --- | --- | --- | --- |
| WF vs Quantitative CT model | 0.023 (-0.030 to 0.076) | 0.3892 | 0.3892 |
| WF vs Clinical model | 0.376 (0.227 to 0.525) | 0.000006 | 0.000017 |
| Quantitative CT model vs Clinical model | 0.353 (0.195 to 0.511) | 0.000044 | 0.000088 |

ΔAUC was calculated as the mean outer-fold AUC of the first model minus that of the second model. Values were based on 50 matched outer-test folds from 10 repetitions of five-fold nested cross-validation. The unadjusted 95% confidence intervals and two-sided P values were obtained using the Nadeau–Bengio corrected resampled *t*-test. Holm correction was applied to the three prespecified comparisons. Positive ΔAUC values favor the first model. All comparisons were exploratory, with DDLPS defined as the positive class.

**Supplementary Table S8. Quantitative calibration performance of the preoperative prediction models**

| Model group | Model | Brier score (95% CI) | Calibration intercept (95% CI) | Calibration slope (95% CI) |
| --- | --- | --- | --- | --- |
| Preoperative benchmark | Clinical | 0.246 (0.224 to 0.269) | -0.270 (-0.413 to -0.096) | 0.454 (-0.328 to 1.301) |
| Preoperative benchmark | Quantitative CT | 0.148 (0.107 to 0.192) | -0.108 (-0.438 to 0.257) | 1.004 (0.669 to 1.626) |
| Regional radiomics | W | 0.153 (0.111 to 0.197) | -0.330 (-0.749 to -0.045) | 1.035 (0.666 to 1.781) |
| Regional radiomics | F | 0.147 (0.103 to 0.195) | -0.229 (-0.465 to 0.089) | 0.605 (0.347 to 1.371) |
| Regional radiomics | S | 0.202 (0.158 to 0.250) | -0.275 (-0.384 to 0.006) | 0.298 (0.236 to 1.282) |
| Regional radiomics | WF | 0.132 (0.087 to 0.179) | -0.165 (-0.563 to 0.161) | 0.885 (0.536 to 1.552) |
| Regional radiomics | WFS | 0.162 (0.117 to 0.210) | -0.263 (-0.490 to 0.007) | 0.616 (0.313 to 1.457) |
| Regional radiomics | WFR | 0.150 (0.108 to 0.195) | -0.232 (-0.626 to 0.031) | 0.955 (0.574 to 1.838) |
| WF-based classifier | LR | 0.132 (0.087 to 0.179) | -0.165 (-0.563 to 0.161) | 0.885 (0.536 to 1.552) |
| WF-based classifier | RF | 0.152 (0.104 to 0.204) | 0.036 (-0.226 to 0.344) | 0.567 (0.395 to 1.163) |
| WF-based classifier | SVM | 0.137 (0.092 to 0.184) | -0.125 (-0.484 to 0.273) | 1.007 (0.688 to 1.578) |
| WF-based classifier | XGB | 0.151 (0.094 to 0.212) | -0.163 (-0.479 to 0.144) | 0.524 (0.380 to 0.885) |

All estimates were calculated using out-of-fold predicted probabilities, with DDLPS defined as the positive class. The 95% confidence intervals were obtained from 5,000 stratified patient-level bootstrap samples. Lower Brier scores indicate better overall probabilistic accuracy; the ideal calibration intercept and slope are 0 and 1, respectively. The WF regional model and the LR classifier represent the same WF-based logistic regression model and therefore have identical results.

**Abbreviations:** CI, confidence interval; DDLPS, dedifferentiated liposarcoma; WF, model using W and F features; LR, logistic regression.

**Supplementary Table S9. Selected features and standardized coefficients of the final WF-based L1-regularized logistic regression model**

| **Feature** | **Feature class** | **Region** | **Standardized coefficient (β)** | **OR per 1-SD increase** |
| --- | --- | --- | --- | --- |
| Intercept | — | — | −0.423 | — |
| Mean | First-order | W | 0.942 | 2.564 |
| Run-length nonuniformity normalized | GLRLM | F | 0.648 | 1.912 |
| Correlation | GLCM | W | −0.457 | 0.633 |
| Short-run emphasis | GLRLM | F | 0.188 | 1.207 |
| Surface-to-volume ratio | Shape | F | −0.109 | 0.897 |
| 10th percentile | First-order | W | 0.058 | 1.060 |
| Maximum 3D diameter | Shape | W | 0.017 | 1.017 |

The table presents the nonzero features retained in the final WF-based L1-regularized logistic regression model refitted using the complete cohort of 97 patients. Radiomics features were standardized before model fitting; therefore, β represents the change in the predicted log-odds of DDLPS per 1-standard-deviation increase in the corresponding feature, and the odds ratio was calculated as exp(β). Positive coefficients indicate higher predicted odds of DDLPS, whereas negative coefficients indicate lower predicted odds. The coefficients from the full-cohort refitted model are provided descriptively and were not independently validated. Abbreviations: W, whole-tumor region; F, CT-defined fat-attenuation subregion; GLRLM, gray-level run-length matrix; GLCM, gray-level co-occurrence matrix; OR, odds ratio; SD, standard deviation; DDLPS, dedifferentiated liposarcoma.

**Supplementary Figure S1. Selection frequencies of regional feature sets and classifiers during selection-aware repeated nested cross-validation.**

The left panel shows the frequencies with which the six candidate regional feature sets were selected, whereas the right panel shows the frequencies with which the four candidate classifiers were selected. Frequencies were calculated across the 50 outer-training folds generated by 10 repetitions of five-fold outer cross-validation. Within each outer fold, regional feature-set and classifier selection was performed exclusively within the three-fold inner cross-validation loop. The selection frequencies reflect the stability of the model-selection procedure and should not be interpreted as measures of diagnostic performance. No single feature set or classifier was selected in a majority of the outer folds, indicating variability among several similarly performing candidate models. **Abbreviations:** W, whole-tumor region; F, CT-defined fat-attenuation subregion; S, CT-defined soft-tissue-attenuation subregion; WF, model using W and F features; WFS, model using W, F, and S features; WFR, model using W, F, and R features; LR, logistic regression; RF, random forest; SVM, support vector machine; XGB, extreme gradient boosting.
